# The Wave Sign Correlates with the Posterior Horn Medial Meniscus (PHMM) Tear in the Anterior Cruciate Ligament (ACL) Deficient Knee

**DOI:** 10.5704/MOJ.2303.013

**Published:** 2023-03

**Authors:** G Gan, DH Toon, WWT Teo, THA Wee

**Affiliations:** Department of Orthopaedics, Khoo Teck Puat Hospital, Singapore

**Keywords:** knee, articular cartilage, anterior-cruciate ligament, meniscus

## Abstract

**Introduction:**

A posterior horn medial meniscus (PHMM) tear subjects the knee to pathological stresses, especially in the setting of a deficient anterior cruciate ligament (ACL). These PHMM tears have to be surgically addressed, however they remain a diagnostic challenge. Hence, this study aims to evaluate the wave sign as an arthroscopic diagnostic aid for the PHMM tear which may be occult.

**Materials and methods:**

This is a retrospective study of 61 consecutive patients (62 ACL-deficient knees) who underwent arthroscopic primary ACL reconstruction between September 2017 and August 2018. We defined PHMM tears as tears located in the posterior one-third of the medial meniscus. Root tears and ramp lesions were included in our analysis. The arthroscopic findings were recorded after a comprehensive arthroscopic survey.

**Results:**

In the sample of ACL-deficient knees, 44 (71.0%) had a concomitant medial meniscus tear. The most common location for the tear was in the posterior horn (81.8%). There were seven occult PHMM tears, not described by the radiologist or identified by the operating surgeon on the pre-operative magnetic resonance imaging. The wave sign was identified in 10 (16.1%) knees, all confirming the presence of the PHMM tear. A positive correlation was found between the presence of the wave sign and the PHMM tear.

**Conclusions:**

The wave sign has a statistically significant but weak positive correlation with the presence of the PHMM. We view the wave sign as a valuable arthroscopic cue to rule-in the presence of the PHMM tear in the ACL-deficient knee.

## Introduction

The menisci in the knee serve a multitude of functions, from load transmission and shock absorption to joint conformity, stability, lubrication and proprioception^1-3^. In the ligamentously compromised knee, the medial meniscus plays an even larger role as a secondary stabiliser to anterior-posterior (AP) translation^4-7^. This, in turn, places significant shear stress on the posterior horn medial meniscus (PHMM), predisposing it to tear^8^.

Meniscal tears have been found to be present in 55-80% of anterior cruciate ligament injuries^9-10^, with an increasing incidence of medial meniscus tears with a delay to surgery^11^. The PHMM tear, in particular the root tears and ramp lesions, may lead to a loss of hoop stresses^12^, increasing contact stresses comparable to a total medial meniscectomy^13-15^. These pathologic stresses predispose to high grade chondral lesions^16^, which may result in further progression to degenerative osteoarthritis^17^. It has been reported that 78% of knees requiring a total knee replacement under the age of 60 had a PHMM tear and had a significant correlation with the severity of osteoarthritis^18^. However, if the PHMM tear were to be repaired anatomically, the abnormal contact stresses would return to normal^15^. It has also been shown to improve functional outcomes, and approximately 80% of patients do not show radiological progression of arthritis at medium term follow-up^19^.

The diagnosis of PHMM tears can be a challenge. Its clinical symptoms are non-specific^20^. Magnetic resonance imaging (MRI) has a false negative rate of 10.8%^21^. Even during arthroscopy, it can be difficult to visualise the posterior medial compartment via the standard anterior portals alone^22,23^. In order to improve the diagnostic yield of PHMM tears that may lie occult, it has been suggested to fenestrate the medial collateral ligament, employ the Gillquist or modified Gillquist maneuver, prior to creating a posteromedial portal^24,25^. As these suggestions are not routine, it will be valuable if arthroscopic cues could aid the decision into performing them.

Few studies have taken effort to establish associated arthroscopic pathologies to assist as a diagnostic aid. Matheny *et al* identified that that medial meniscus root tears were associated with articular cartilage defects^26^, and Zhang *et al* had recognised a characteristic chondral lesion termed the wave sign to suggest the presence of the ramp lesions^27^. Hence, this study aims to evaluate the wave sign as an arthroscopic diagnostic aid for the PHMM tear which may be occult.

## Materials and Methods

This is a retrospective study that was conducted on consecutive patients who underwent arthroscopic primary ACL reconstruction. These surgeries were performed by two sports surgeons from the institution between September 2017 and August 2018 inclusive.

The inclusion criteria for this study were patients 16 years and older with a MRI diagnosis and arthroscopic confirmation of an ACL rupture, who underwent arthroscopic primary ACL reconstruction. This was regardless of the meniscal procedures performed in the same setting. Exclusion criteria were patients with multi-ligamentous knee injuries, patients undergoing revision surgery, or patients with incomplete MRI or arthroscopic images. Institutional Review Board approval was obtained prior to conducting this study.

The study patients were identified using the institution’s electronic database. Patient demographics, pre-operative MRI findings, and intra-operative data were retrieved from their electronic records. Their MRI images were reviewed, alongside their arthroscopic images.

**Fig. 1:**
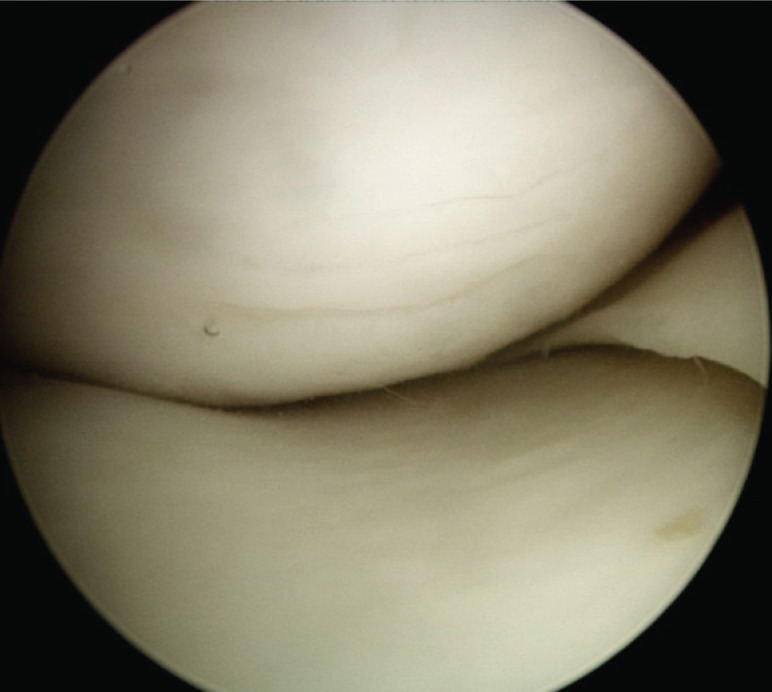
The Wave Sign (arrow); Characteristic wave-like chondral lesions oriented transversely over the weightbearing surface of the medial femoral condyle of the right knee.

We defined PHMM tears as tears located in the posterior one-third of the medial meniscus, inclusive of root tears and ramp lesions. Also, we defined the occult tear as tears not previously described by the radiologist or identified by the operating surgeon on the pre-operative MRI. As for the characteristic wave sign, we reference Zhang *et al* description of transversely oriented wave-like chondral lesions over the weightbearing surface of the medial femoral condyle (Fig. 1)^27^. These findings were recorded after a comprehensive arthroscopic survey including a trans-notch view, with care not to cause iatrogenic cartilage lesions.

The chi-square test was used for the statistical analysis. Spearman’s correlation was used to assess the relationship between wave sign and the PHMM tear. A p-value of <0.05 was considered statistically significant.

## Results

There were a total of 61 patients, with a mean age of 25.3 (range 17-46) years of age, who were included in this study. This patient sample had a gender distribution of 49 males: 12 females, with a multi-ethnic make up of 22 Chinese, 33 Malay, 4 Indian, 1 Others (Table I). Of these 61 patients, there was 1 patient who had undergone arthroscopic ACL reconstruction of both knees at different time points within the defined study period.

**Table I:** Patient demographics.

Sample Size (n)	61
Age (years)	25.3 (range 17-46)
Gender	
Male	49
Female	12
Ethnicity	
Chinese	22
Malay	33
India	4
Others	1

For the 62 ACL-deficient knees that were reviewed, 48 (77.5%) knees had concomitant meniscal tears. A total of 22 (35.5%) had tears in both menisci, 22 (35.5%) had a tear of the medial meniscus only, 4 (6.5%) had a tear of the lateral meniscus only. Only 14 (22.5%) had no concomitant meniscal tears (Table II). Of the 44 of medial meniscus tears, 36 (81.8%) were located in the posterior horn. A total of 22 were located in the posterior horn alone, and 14 were in the posterior horn with an extension into the meniscus body. There were seven occult PHMM tears, which was not described by the radiologist or identified by the operating surgeon on the pre-operative MRI.

**Table II:** Distribution of meniscal tears in the ACL-deficient knee.

No. of ACL-deficient knees 9n)	62
Both menisci torn	22 (35.5%)
Medial meniscus tear only	22 (35.5%)
Lateral meniscus tear only	4 (6.5%)
No concomitant meniscal tears	14 (22.5%)

The wave sign was identified in 10 of the 62 knees (16.1%). They were all characterised as transversely oriented wave-like chondral lesions over the weightbearing surface of the medial femoral condyle. All 10 wave-sign positive knees had a PHMM tear. A positive correlation was found between the presence of the wave sign and the PHMM tear (Table III). It was statistically significant with a Spearman correlation coefficient of r = 0.385 (p-value = 0.02), with a Fischer exact test statistic of 0.004. The wave sign had a sensitivity and specificity of 27.8% and 100%, with a positive predictive value (PPV) and negative predictive value (NPV) of 100% and 50.0% for diagnosis of the PHMM tear, respectively. However, a positive correlation was not found when the wave sign was correlated specifically with the presence of the occult PHMM tear not picked up on MRI (Table IV). The Spearman correlation coefficient was r = 0.121 (p-value = 0.350), with a Fischer exact test statistic of 0.314. The wave sign had a sensitivity and specificity of 25.6% and 85.5%, with a PPV and NPV of 20.0% and 90.4% for the diagnosis of the occult PHMM, respectively.

**Table III:** Correlation of the wave sign with the PHMM tear in the ACL-deficient knee.

			PH MM Tear	
		Yes	No	Total
Wave Sign	Yes	10	0	10
No	26	26	52	
Total	36	26	62	
Sensitivity		27.8%		14.2% - 45.2%
Specificity		100.0%		86.6% - 100.0%
Positive Predictive Value		100.0%		n/a
Negative Predictive Value		50.0%		45.0% - 55.1%

**Table IV:** Correlation of the wave sign with the occult PHMM tear in the ACL-deficient knee.

			Occult PH MM Tear	
		Yes	No	Total
Wave Sign	Yes	2	8	10
No	5	47	52	
Total	7	55	62	
Sensitivity		28.5%	3.67% - 71.0%	
Specificity		85.5%	73.3% - 93.5%	
Positive Predictive Value		20.0%	6.17% - 48.72%	
Negative Predictive Value		90.4%	85.3% - 93.8%	

## Discussion

The PHMM tear is common in the ACL-deficient knee. In this study, it was found that 71.0% of ACL-deficient knees had a concomitant medial meniscus tear. Of these medial meniscus tears, 81.8% of them were located in the posterior horn, inclusive of the root tears and ramp lesions. These statistics are well supported in the published literature^9,10^. However, PHMM tear remains a diagnostic challenge with the clinical and radiological limitations. Even arthroscopic evaluation is not without its challenges, whereby lesions may not be missed even with a trans-notch view using a 70° arthroscope via the standard anterior ports^22,28^. Bumberger *et al* has gone on to suggest that an additional posteromedial portal should be considered the gold standard in diagnosing ramp lesions^23,29^, though this is not currently routine.

The wave sign incidence in this study’s sample was 16.1%. This is a substantially higher percentage when compared to the 4.9% (78/1596) in Zhang *et al*^27^. This difference is likely attributable to the difference in the study sample’s arthroscopy indication - primary ACL reconstruction as compared to all indications, respectively. All 10 wave sign positive knees had PHMM tears. This arthroscopic sign was found to positively correlate, albeit weakly, with the presence of the PHMM tear. This was similar with Zhang *et al’s* results, by which all patients with the positive wave sign had a confirmed ramp lesion^27^. Our study’s statistical assessment for the wave sign, with a specificity of 100% and a PPV of 100%, makes it an attractive rule-in sign for the presence of the PHMM tear.

The suggested pathophysiology for the chondral wave sign is likely the result of the (i) AP translational instability, and (ii) high contact shear forces on the weight bearing surface femoral condyle. In the ACL-deficient knee with a concomitant PHMM tear, AP translational instability results in abnormal joint kinetics^30,31^. Compounded by the presence of the PHMM tear, which in turn disrupts the hoop stresses, decreasing contact area and increasing contact stresses, the chondral surface is failure under tension over time^5,16,32,33^. The recognition of arthroscopic cues to evaluate for and treat the PHMM tear will aid the goal of joint preservation, and delay the degenerative process^15,19^.

However, there may be wave sign mimics. One such example is the iatrogenic lesions caused by arthroscopic instruments. It has been found that the incidence of iatrogenic lesions to be 4% - 10%^34^. Though these lesions tend to take on a regular linear appearance and have a more sagittal-based orientation along the trajectory of the arthroscopic instruments. This is as opposed to the wave sign, which has a wave-like irregular appearance, with a transverse orientation over the weightbearing surface of the medial femoral condyle.

## Conclusion

We recognise that there are some limitations of this study. This is a retrospective study with a small sample size. The identification of the wave sign can have a verification bias, though it has been recognised arthroscopic feature in our institution prior to the study period and had been evaluated for by two sports surgeons independently. In conclusion, we view the wave sign as a valuable arthroscopic cue to rule-in the presence of the PHMM tear in the ACL-deficient knee.
